# Lncident: A Tool for Rapid Identification of Long Noncoding RNAs Utilizing Sequence Intrinsic Composition and Open Reading Frame Information

**DOI:** 10.1155/2016/9185496

**Published:** 2016-12-27

**Authors:** Siyu Han, Yanchun Liang, Ying Li, Wei Du

**Affiliations:** ^1^College of Computer Science and Technology, Key Laboratory of Symbol Computation and Knowledge Engineering of Ministry of Education, Jilin University, Changchun 130012, China; ^2^Zhuhai Laboratory of Key Laboratory of Symbol Computation and Knowledge Engineering of Ministry of Education, Zhuhai College of Jilin University, Zhuhai 519041, China

## Abstract

More and more studies have demonstrated that long noncoding RNAs (lncRNAs) play critical roles in diversity of biological process and are also associated with various types of disease. How to rapidly identify lncRNAs and messenger RNA is the fundamental step to uncover the function of lncRNAs identification. Here, we present a novel method for rapid identification of lncRNAs utilizing sequence intrinsic composition features and open reading frame information based on support vector machine model, named as Lncident (LncRNAs identification). The 10-fold cross-validation and ROC curve are used to evaluate the performance of Lncident. The main advantage of Lncident is high speed without the loss of accuracy. Compared with the exiting popular tools, Lncident outperforms Coding-Potential Calculator, Coding-Potential Assessment Tool, Coding-Noncoding Index, and PLEK. Lncident is also much faster than Coding-Potential Calculator and Coding-Noncoding Index. Lncident presents an outstanding performance on microorganism, which offers a great application prospect to the analysis of microorganism. In addition, Lncident can be trained by users' own collected data. Furthermore, R package and web server are simultaneously developed in order to maximize the convenience for the users. The R package “Lncident” can be easily installed on multiple operating system platforms, as long as R is supported.

## 1. Introduction

Long noncoding RNAs (lncRNAs) are transcripts of greater than 200 nucleotides without evident protein-coding function [1]. LncRNAs widely exist in many species ranging from invertebrata to mammals, such as* Caenorhabditis elegans* (*C. elegans*) [2],* Arabidopsis* [3], mouse (*Mus musculus*) [4], and humans (*Homo sapiens*) [5].

LncRNAs have been found to play critical roles in the regulation of gene expression at the different levels of transcription, posttranscription, translation, histone modifications [6], and DNA methylation [7]. Most of the lncRNAs are important regulators of gene expression. Human genome contains many thousands of lncRNAs [8]. Some studies have proved that lncRNAs dysfunctions are associated with multiple diseases such as cancer, cell fate determination, and pathogenesis of human disease [9]. The experimentally supported relations between lncRNAs and diseases are manually collected in the databases lncRNADisease [9] and Lnc2Cancer [10].

The fundamental step of uncovering the function and mechanism of lncRNAs is to identify lncRNAs and protein-coding RNAs. Currently, many tools have been developed to distinguish lncRNAs from protein-coding RNAs. Coding-Potential Calculator (CPC) [11] and Coding-Potential Assessment Tool (CPAT) [12] are the tools used to assess the coding potential of the nucleotide sequences. CPC and CPAT all utilize the sequence-based features of nucleotide composition and codon usage. In addition, CPC aligns the sequences against protein database. The Fickett TESTCODE score [13, 14] and Hexamer score are used in CPAT. The Coding-Noncoding Index (CNCI) [15] and PLEK (predictor of long noncoding RNAs and messenger RNAs based on an improved *k*-mer scheme) [16] are the tools to predict the sequences according to intrinsic composition, which are alignment-free. LncRNA-MFDL [17] integrates the minimum energy with some features of CNCI and PLEK and finally uses deep learning algorithm to build the classifier.

CPC is an alignment-based method. The main disadvantage of CPC is the high time cost. Other tools also have some minor disadvantages. The cutoff of CPAT's new-trained model is needed to be determined by users. CNCI shows unbalanced results between sensitivity and specificity. And the performances of PLEK fluctuate on different species. Besides, almost all the above tools have relatively poor identification results on invertebrata or microorganism. Currently, only CPC and CPAT provide the web servers. For the local use, all these tools need a UNIX/Linux platform, which limits their availability. Thus, we hope to present a tool that achieves high accuracy efficiently but also easy to use. In this paper, we developed an alignment-free tool, named as “Lncident” (*Lnc*RNAs* ident*ification), to rapidly predict lncRNA and messenger RNA (mRNA) utilizing sequence intrinsic composition features and open reading frame information based on support vector machine (SVM). In order to validate Lncident, the existing popular tools CPC, CPAT, CNCI, and PLEK are used to compare with our proposed “Lncident” on different species, including human, mouse,* C. elegans,* and* S. cerevisiae*. The 10-fold cross-validation (10-fold CV) and Receiver Operating Characteristic (ROC) curve show that Lncident has high accuracy. On human dataset, Lncident (sensitivity: 0.9535, specificity: 0.9795, and accuracy: 0.9665) outperforms several popular tools such as CPC (sensitivity: 0.6625, specificity: 0.9992, and accuracy: 0.8309), CPAT (sensitivity: 0.9160, specificity: 0.9848, and accuracy: 0.9504), CNCI (sensitivity: 0.9702, specificity: 0.9157, and accuracy: 0.9430), and PLEK (sensitivity: 0.7622, specificity: 0.9507, and accuracy: 0.8565). In addition, Lncident can complete the identification process in several minutes while CPC needs dozens of hours and CNCI need tens of minutes. Furthermore, Lncident achieved the best identification performance on* S. cerevisiae* compared with other tools. Therefore, the characteristic of Lncident is embodied in keeping lower time complexity and high accuracy at the same time. In addition, to greatly facilitate the users for different demands such as local use and online use, an R package and a web server of Lncident are developed at the same time. The R package “Lncident” is available at http://csbl.bmb.uga.edu/mirrors/JLU/Lncident/index.php, which is applicable for multiple operating system platform. The web server of Lncident can be accessed by http://csbl.bmb.uga.edu/mirrors/JLU/Lncident/annotate.php. Web interface is a practical and effective alternative for small-scale identification. Lncident can be trained on users' own datasets as well, which greatly facilitates the researches who are focusing on some poor-explored species.

## 2. Materials and Methods

**Figure 1:**
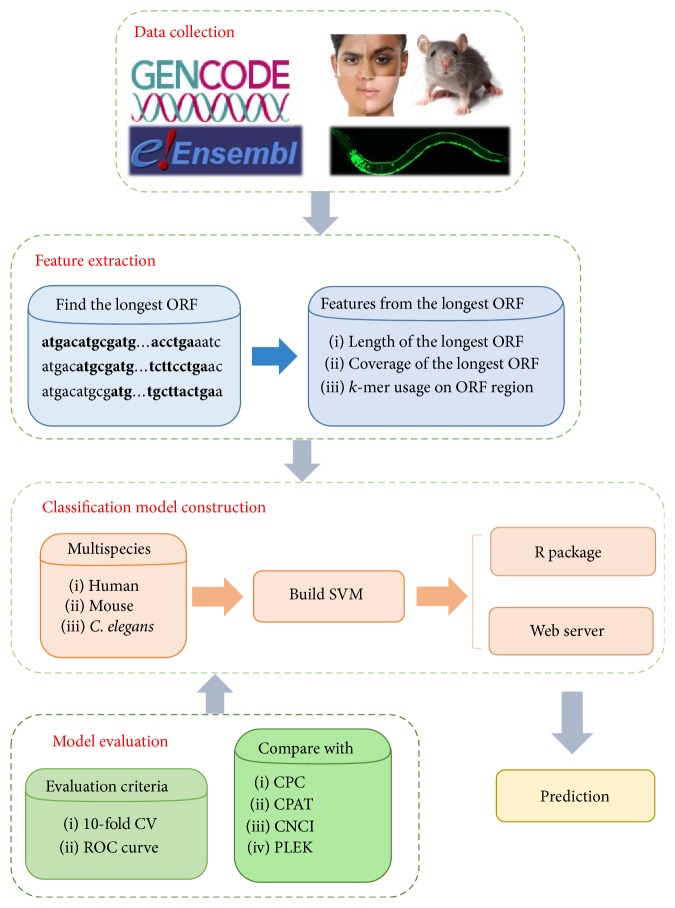
The framework of Lncident: containing data collection, feature extraction, classification model construction, and model evaluation.

The main steps of Lncident contain data collection, feature extraction, classification model construction, and model evaluation. An overall procedure is displayed in Figure 1.

Firstly, mRNA and all the lncRNA sequences of the different species, including human, mouse,* C. elegans,* and* S. cerevisiae*, are collected from GENCODE and Ensembl database. Next, the intrinsic composition and open reading frame features are extracted. Then, the classification model based on SVM are constructed for different species. Lncident is evaluated by performing 10-fold CV and ROC curve. Finally, in order to facilitate the users, the R package and the web server of Lncident are developed at the same time, which are freely available.

### 2.1. Data Collection and Description

Two human datasets are used to train and test the tools. The first human dataset is collected from GENCODE 24 (GRCh38.p5) [18–20], which contains 28,031 lncRNAs and the same amount of protein-coding transcripts. The other one is obtained from [21]. The model for mouse is trained and tested on the dataset of GENCODE M9 (GRCm38.p4) [22], which contains 13,046 lncRNAs and 13,046 protein-coding transcripts. Because of the insufficiency (only 1,638) of lncRNAs, the model for invertebrata is trained and tested on* C. elegans* datasets containing 22,929 noncoding RNAs (ncRNAs) and 22,929 coding sequences (CDs) randomly selected from Ensembl [23] in training set and 2,000 ncRNAs and 2,000 CDs in test set. To test the performance of the model of* C. elegans*, dataset of* S. cerevisiae* is collected from Ensembl database [24–26], which contains 413 ncRNAs and 1,500 CDs. Detailed information of datasets has been summarized in Supplementary Table S1 (see Supplementary Material available online at http://dx.doi.org/10.1155/2016/9185496).

### 2.2. Features Extraction

The extracted features of Lncident can be divided into two categories: open reading frame (ORF) features and *k*-mer adjoining-base(s) features from sequence intrinsic composition. ORF is one of the most general but significant criteria for identifying the coding and noncoding sequences, which is because protein-coding sequences tend to possess a relatively long ORF. LncRNAs may also have some ORFs but ordinarily very short. Thus, the first feature is the length of ORF. For one sequence, we firstly find all the ORFs in three forward frames and then take the maximum length as the ORF length of this sequence. The second feature is the ORF coverage, which is defined as the ratio of the maximum length of ORF to sequence length.

**Figure 2:**
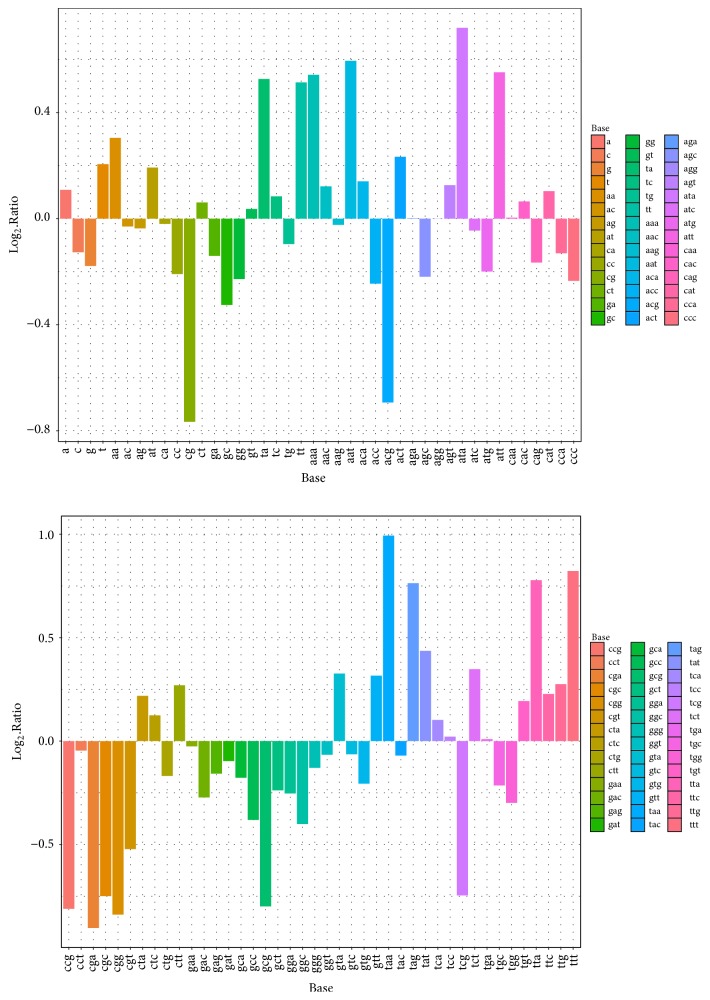
The distribution of the adjoining base(s) on lncRNAs and coding sequences.

Next the *k*-mer adjoining-base(s) features can be computed as follows:

(1) k-mer  adjoining  bases  frequencies=cil−k+1,k=1,2,3,4,5;  i=1,2,…,4k,

where *k* means the *k* base(s) combined together; *c* denotes the occurrence of one kind of adjoining-base(s) combination; and *l* is the length of the longest ORF; hence, the denominator means the total occurrences of all *k* adjoining-base(s) combinations in this region. When *k* = 1, there will be A, C, G, and T, 4 adjoining-base combinations, and when *k* = 2, there will be AA, AC, AG,…, TC, TG, TT, 16 combinations. The adjoining-base(s) features are, therefore, 4 + 16 + 64 + 256 + 1024 = 1364 frequencies of the adjoining-base(s). Then, we analysed the adjoining-base(s) bias of human in lncRNAs and coding sequences (CDs). In Figure 2, the log_2_-ratio is used to show the difference of the distribution of the adjoining base(s) between human lncRNAs and CDs. The log_2_-ratio can be obtained from the following formula:

(2) log⁡2-Ratio=log⁡2⁡adjoining  bases  frequencies  of  lncRNAs adjoining  bases  frequencies  of  CDs.

Figure 2 shows the obvious differences between the distribution of lncRNAs and CDs. In addition, the log_2_-ratio of frequencies of *k*-mer adjoining base(s) between human lncRNAs and protein-coding genes are calculated (see Supplementary Figure S1). We can notice that Supplementary Figure S1 is somewhat different from Figure 2. In Figure 2, the log_2_-ratios of bases combination such as AC, AG, and ATC are all negative numbers while in Figure S1 they are all positive. In addition, the log_2_-ratio of TAA nearly reaches 1.0 in Figure 2 while in Figure S1 it is even less than 0.5. There are many other noticeable differences between these two figures, which indicates that the untranslated regions (UTRs) of protein-coding transcripts indeed affect the performance of the classifier. UTRs make protein-coding gene look like noncoding sequence, which may lead to a wrong classification for protein-coding sequence. The *k*-mer adjoining-base(s) features in Lncident are calculated in ORF region, which is different from the improved *k*-mer scheme of PLEK. PLEK calculates the nucleotides compositions on the whole sequence, and *k*-mer frequencies are also multiplied with weights in order to scale the data. By calculating the combinations frequencies on ORF region, we can maximally reduce the side effect of UTRs of mRNAs. The figures are drawn by employing the R package “ggplot2” [27].

In addition, in terms of ROC curve (see Supplementary Figure S2), the size of *k* is determined. With the increase of *k*, the performance of *k*-mer feature group also gets better. Nonetheless, the values of area under the curve (AUC) for *k* = 5 and *k* = 6 are equal. The performance of *k* = 6 is not significantly better than *k* = 5. But *k* = 6 means 4,096 features, which takes lots of time to calculate the frequencies. Therefore, here take *k* = 5.

**Figure 3:**
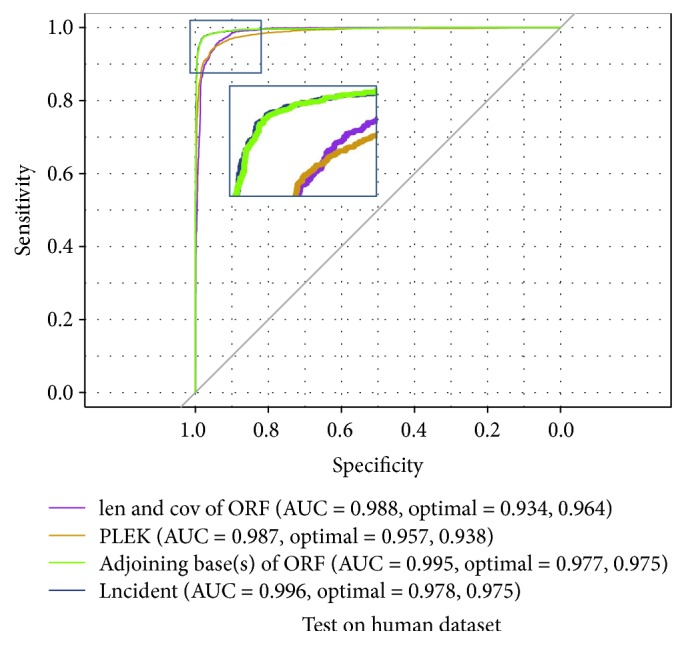
The Receiver Operating Characteristic (ROC) curve of several feature categories.

Finally, we drew a ROC curve to further compare the classification performances of four different feature groups (see Figure 3): the length and coverage of the longest ORF and improved *k*-mer scheme (PLEK), adjoining-base(s) features, and Lncident, which is the combination of the first and the third group. Lncident achieved the best performance. Compared with the features in PLEK, the features in Lncident show the significant improvement. RFE (recursive feature elimination) was also conducted on different feature groups (see Supplementary Figure S3) in order to assess whether all the feature groups are necessary. Based on SVM-RFE algorithm, we also calculated the important score of each feature, and the top 10 important features are displayed in Table S2 (see Supplementary File). The most important feature group, as we anticipated, is ORF feature group. Except ORF features, the other top 10 important features are all related to nucleotides “C” and “G.”

### 2.3. Classification Model Construction

The open reading frame features and adjoining features are incorporated into a support vector machine (SVM) model to construct a classification model. Utilizing the datasets of human, mouse, and* C. elegans*, we constructed lncRNA and mRNA classifiers by employing the LIBSVM of “e1071” package in R [28], where the standard radial basis function kernel (RBF kernel) is used and all the features are scaled.

## 3. Evaluation

We evaluated Lncident by comparing with CPC, CPAT, CNCI, and PLEK, which are the popular and representative tools of lncRNA prediction. LncRNA-MFDL is not involved in our comparison because the download link of lncRNA-MFDL software provided in [17] is forbidden. In our evaluation, lncRNAs are labelled as positive class while protein-coding transcripts or CDs are labelled as negative class; then, sensitivity, specificity, accuracy, *F*-measure, Matthews correlation coefficient (MCC), and Kappa value [29] are used to assess the performances of Lncident:

(3) Sensitivity=TPTP+FN,Specificity=TNTN+FP,Accuracy=TP+TNP+N,Precision=TPTP+FP,F-measure=2×Sensitivity×PrecisionSensitivity+Precision,MCC=TP×TN−FP×FNTP+FPTP+FNTN+FPTN+FN,k=Pr⁡a−Pr⁡e1−Pr⁡e.

### 3.1. Performance on GENCODE Database

**Figure 4:**
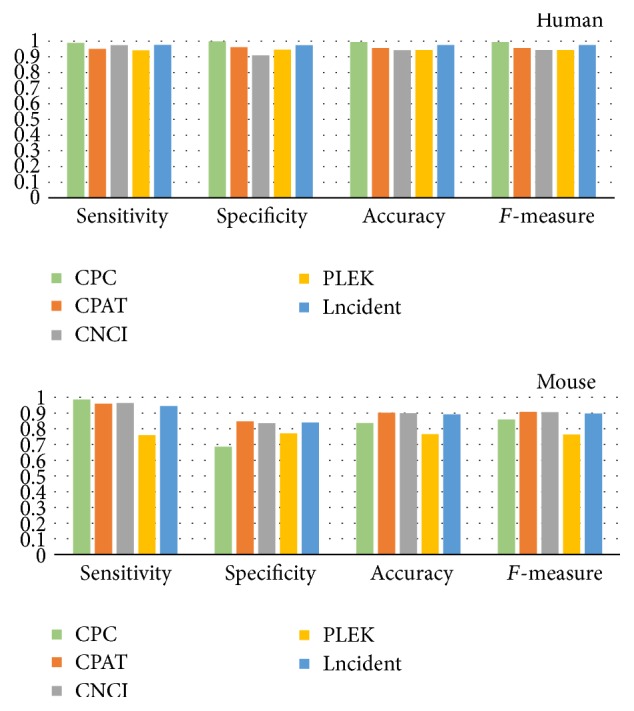
The performances of different tools on different species.

During the comparison of the tools, we found that different test sets led to fluctuated performances and it is unfair to compare these tools by testing the classifiers only using some randomly selected sequences. Hence, Lncident will firstly be evaluated by performing 10-fold CV and each test set for Lncident will be also used to test other four tools. In addition, in order to have a more unbiased and relatively fair results, each tool's most appropriate species model will be selected when conducting the tests. CPAT web server has the model for mouse (GRCm38/mm10), which is also the assembly of our mouse dataset. CNCI only have the models for vertebrata and plantae; hence, vertebrata model was selected definitely. We tested CPC by using its web server because a massive-scale prediction on local version is time-consuming. Note that Lncident undertook the 10-fold CV and the test set has no identical sequence with the training set. The final performance of five tools is the average of all the test sets results (see Supplementary Tables S2 and S3). The overall performances are displayed in Figure 4.

From Figure 4, we can find that Lncident achieved a balanced overall result with high accuracy. Lncident is only slightly inferior to CPC on human dataset, but CPC, due to alignment process, needs several hours to complete the identification which can be finished by Lncident in a few minutes. The main advantage of Lncident over other tools is high speed without the loss of accuracy, which meets the needs for high-through sequencing and is suitable for lncRNA identification at large scale.

The constructed model for human can be applied to many other species, especially mammals, such as chimpanzee (*Pan troglodytes*) or Gorilla (*Gorilla gorilla gorilla*). But we specially built a classifier for mouse, because mouse is one of the most studied species for lncRNA analysis except the human. The quantity of annotated lncRNAs in mouse database only amounts to a half of lncRNA in human database, which leads to all the tools having a decline in performance. The experiment validated that lncRNAs for other species are even less. Therefore, the classifiers constructed for other species may present less favourable outcomes than the models for human.

### 3.2. Performance on Human Dataset 2

According to our test on human datasets collected from GENCODE, we can find that CPC displayed the best result. Nonetheless, CPC, as an alignment-based tool, will align the sequences against the whole protein reference database and CPC greatly depends on its reference protein database. It is not surprising that CPC will obtain an excellent result when the sequences are selected from database. Here, we utilized the datasets from [21] to retrain Lncident, CPAT, and PLEK. Then, all the tools' performances are evaluated on new test sets. The training and testing datasets have no transcripts from the same genes and only one transcript from each gene is collected in training set. Since CPC and CPAT provided web interface, both stand-alone and web server version are tested. UniRef90 [30], as CPC suggested, was selected as reference protein database for CPC in our test. Note that the prebuilt model of other tools may have the sequences overlapping with the test sets, which made these tools have some advantages over Lncident and other tools with new-trained model. The results are displayed in Table 1.

CPC achieved the highest specificity but had a poor performance on sensitivity. Lncident presents the most balanced performance, even compared with other tools' default model. Lncident also obtained the highest accuracy and *F*-measure. The performances of web server and local version of CPC and CPAT show some minor differences which may result from the different genome assembly of the training set.

### 3.3. Performance on Invertebrata Dataset

Due to the extremely limited amount of experimental validated ncRNAs in invertebrata/microorganism, there is no specific tool to predict the ncRNAs in these species. The existing tools including CNCI and PLEK all utilize the model trained on human database to predict the sequences of invertebrata or microorganism. The predicted results for invertebrata/microorganism are usually not satisfactory. We here tested all the tools on* C. elegans* dataset with their default model. For our proposed method Lncident, we also made this experiment using the classifier model trained on human database (see Table 2). CPC and CPAT were tested on their web servers, since web server generally presents better results.

For the sequences selected from database, CPC still had the best performance. For other alignment-free tools, Lncident had the best performance on every aspect. Then, we trained Lncident, CPAT, and PLEK with dataset of* C. elegans*. We evaluated Lncident's new model with 10-fold CV and Lncident achieved a satisfactory result (sensitivity: 0.9970, specificity: 0.9955, accuracy: 0.9962, and *F*-measure: 0.9962). From Table 2, we noticed that both CPAT and Lncident surpassed CPC. However, users have to determine a cutoff for CPAT, and the best cutoffs of different species vary widely. For example, the cutoff of human is 0.364, while the best cutoff for* C. elegans* is 0.850 in our research. Lncident is marginally 0.001 lower than CPAT on accuracy, but no cutoff needs to be chosen by users. Moreover, the model provided by Lncident for invertebrata can save users training models by themselves.

Next, we evaluated each tool's default model on dataset of* S. cerevisiae* (see Table 3). We can notice that CPC, despite having a long wait, struck a good balance between sensitivity and specificity, obtaining a reasonable result. Nonetheless, all the alignment-free tools presented a sharp decline on specificity. Though Lncident still outperformed other alignment-free tools, these disappointing performances on* S. cerevisiae* indicated that it is necessary to build a specific classifier model for microorganism. However, the annotated noncoding transcripts of microorganism are too few to ensure a better classifier. Thus, we again evaluated Lncident, CPAT, and PLEK by utilizing the model trained on* C. elegans*.

For Lncident with new-trained model, it is apparent that overall accuracy and *F*-measure are significantly enhanced. According to Table 3, Lncident.train presented the best result with a much higher speed than CPC and CNCI. As we mentioned above, the cutoff of model trained on* C. elegans* of CPAT is 0.850. However, the cutoffs of species are diverse dramatically and cannot be applied to other species instantly. We cannot guarantee that 0.850 is the appropriate cutoff for other species. Even worse, not every species has sufficient information for users to determine the best cutoff. When we use the model trained by other species, Lncident achieves a more satisfying result. Compared with other tools, Lncident provides more choice for the users as well.

### 3.4. Running Time

We evaluated the running time of these tools on human dataset 2 which contains 4,000 mRNAs and 4,000 lncRNAs (Table 3). We can notice that CPAT is the most efficient tool mainly because CPAT is based on logistic model which is much faster than SVM. Since CPC is an alignment-based tool, it needs lots of time to align the sequences against the reference protein database. The script of Lncident is written in R which is slower than Python and C, but thanks to parallel computing, the speed of Lncident is comparable to PLEK (which is written in Python), and much faster than CPC and CNCI. All tools were tested on the same PC with 3.40 GHz Intel processor, 8 GB memory, and Linux operating system (Table 4).

## 4. R Package

For the user's convenience, an R package “Lncident” is developed and can be download from http://csbl.bmb.uga.edu/mirrors/JLU/Lncident/index.php. The Lncident R package can be easily installed. And the reference manual document is also available on the above website. The R package Lncident provides an option for the users to train a classifier on users' own datasets, which greatly facilitates the researches who are interesting on some poor-explored species. Currently, Lncident provides three functions: extract_features(), lnc_pred(), and find_orfs(). The first function can extract the features when users want to build their own model and the second function is used to predict the sequences utilizing the default model. Lncident can also act as an offline ORFs finder; the third function can help to find all the ORFs in the sequences.

Lncident depends on the packages: “seqinr” [31] and “e1071” [32]. During the development, we also used the package “roxygen2” [33] to facilitate our work. The code of finding the ORFs is inspired by Avril Coghlan's R code [34]. For further information, please refer to the documentation of Lncident.

## 5. Web Server

**Figure 5:**
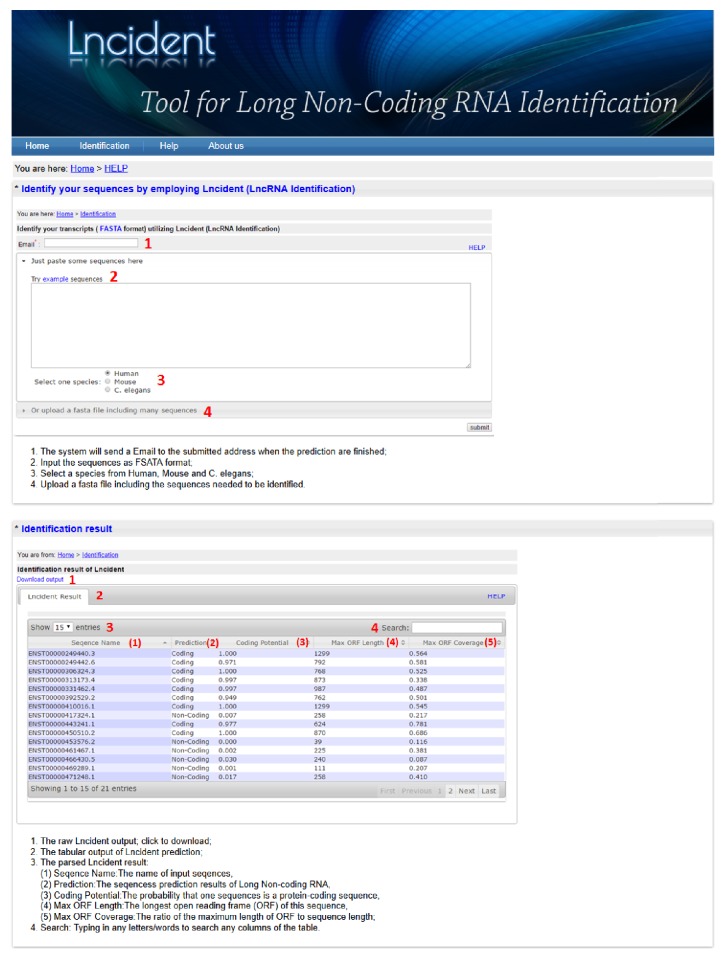
The screenshots of Lncident input and output.

In order to maximize the convenience for the users, a user-friendly web interface for Lncident (http://csbl.bmb.uga.edu/mirrors/JLU/Lncident/annotate.php) also is developed. The input and output description of Lncident are displayed in Figure 5.

The input of Lncident web server is FASTA sequences, which can be directly uploaded from the input box or from a local FASTA sequence file. Users can also select different models which are trained on different species (human; mouse and* C. elegans*).

When the identification is processing, users can bookmark this page and come back later. Or users can also choose to stay at this page, which will be automatically refreshed every 5 seconds. The results will be shown in the browser when the prediction is finished. In addition, when the submitted job is at large scale, the user can input an email address. If an email address is left, the user will be notified by an email including a link once the job is finished. Using this link, the results can be easily retrieved from the web server.

## 6. Conclusion

With the rapid development of the next-generation sequencing technology and the more discoveries of the importance of noncoding RNAs' functions, it is of great importance to identify noncoding RNAs from protein-coding ones with fast speed and high accuracy. More and more unidentified transcripts are obtained from various sequencing platforms, and it seems highly improbable that these transcripts would be put into the database in advance. Therefore, the discriminative power of alignment-based tools will be impaired when dealing with practical problems. Utilizing sequence intrinsic composition features and open reading frame information based on machine learning model (support vector machine), we propose a novel method for rapid identification of lncRNAs named as Lncident (LncRNAs identification). For the users' convenience, a user-friendly R package and web server are developed at the same time, which can be freely available. Compared with other tools, through 10-fold cross-validation and test on different species databases, Lncident shows the higher speed and better performances.

The existing tools including CNCI and PLEK all utilize the classifier models trained on human database to directly predict the sequences of other species. In this paper, in order to maximize to meet the practical demand, we trained the different classifier models for the human, mouse, and* C. elegans* species. The specific classifier model construction of Lncident for mouse is because the mouse is one of the most studied species for lncRNA analysis except the human. Furthermore, there is no specific tool to predict the ncRNAs in microorganism. The predicted results for microorganism are usually not satisfactory. But due to the extremely limited amount of experimental validated ncRNAs in microorganism, it is unpractical to directly construct a classifier model by training on microorganism. Therefore, we carefully choose* C. elegans *to train a classifier model to identify ncRNA of invertebrata/microorganism. The test on the sequences of microorganism* S. cerevisiae* shows that Lncident can achieve the best prediction performance. Lncident provides a great potential and application prospect for the microorganism. The Lncident can be tailored to multiple species by building a new model on users' own datasets.

## Supplementary Material

This Supplementary Material contains additional information of the original article. In the Supplementary File, a detailed description of datasets and a close comparison of different tools' performances on human/mouse datasets are presented. In addition, the performances of original k-mer scheme (k from 1 to 6) and the performances of different feature group combinations are also included in this material. Finally, we provided top 10 features of Lncident and each feature's importance score, please refer to the Table S2 in Supplementary Material.

## Figures and Tables

**Table 1:** The performances on human dataset 2.

Tools	Sensitivity	Specificity	Accuracy	*F*-measure	MCC	Kappa
CPC.local	0.6613	**1**	0.8306	0.7961	0.7028	0.6612
CPC.web	0.6625	0.9992	0.8309	0.7966	0.7028	0.6618
CPAT.local	0.9333	0.9802	0.9568	0.9557	0.9145	0.9135
CPAT.web	0.9535	0.9673	0.9604	0.9601	0.9208	0.9208
CNCI	0.9702	0.9157	0.9430	0.9445	0.8873	0.8860
PLEK	**0.9952**	0.8918	0.9435	0.9463	0.8918	0.8870
CPAT.train^*∗*^	0.9160	0.9848	0.9504	0.9486	0.9029	0.9008
PLEK.train^*∗*^	0.7622	0.9507	0.8565	0.8416	0.7260	0.7130
Lncident	0.9535	0.9795	**0.9665**	**0.9661**	**0.9333**	**0.9330**

Lncident displayed a satisfying overall performance. CPC and CPAT were tested on stand-alone version and web server. ^*∗*^The suffix of “train” means CPAT and PLEK with the new-trained model.

**Table 2:** Comparisons of *C. elegans*.

Tools	Sensitivity	Specificity	Accuracy	*F*-measure	MCC	Kappa
CPC.web	0.9990	**0.9895**	**0.9942**	**0.9942**	**0.9885**	**0.9884**
CPAT.web	0.9910	0.9187	0.9548	0.9564	0.9120	0.9097
CNCI	0.9938	0.7744	0.8841	0.8955	0.7873	0.7681
PLEK	0.9987	0.4526	0.7256	0.7845	0.5387	0.4513
Lncident^*∗*^	**1**	0.9545	0.9772	0.9778	0.9555	0.9545
CPAT.train^*∗∗*^	**0.9995**	0.9950	**0.9972**	**0.9973**	**0.9945**	**0.9945**
PLEK.train^*∗∗*^	0.9795	0.9950	0.9872	0.9872	0.9746	0.9745
Lncident.train^*∗∗*^	0.9950	**0.9975**	0.9962	0.9962	0.9925	0.9925

For tools with default models, Lncident presented the best result among the alignment-free methods. Both Lncident and CPAT outperformed CPC by utilizing new-trained model. ^*∗*^Lncident with model trained on human. ^*∗∗*^The suffix of “train” means the tools with model trained on *C. elegans*.

**Table 3:** Comparisons of *S. cerevisiae*.

Tools	Sensitivity	Specificity	Accuracy	*F*-measure	MCC	Kappa
CPC.web	0.9734	**0.9687**	**0.9697**	**0.9327**	**0.9145**	**0.9132**
CPAT.web	**1**	0.7980	0.8416	0.7316	0.6785	0.6304
CNCI	0.9758	0.6760	0.7407	0.6190	0.5377	0.4598
PLEK	0.9903	0.5507	0.6456	0.5468	0.4491	0.3407
Lncident^*∗*^	**1**	0.8620	0.8918	0.7996	0.7578	0.7295
CPAT.train^*∗∗*^	**1**	0.9487	0.9597	0.9147	0.8942	0.8886
PLEK.train^*∗∗*^	0.9758	0.8053	0.8421	0.7274	0.6682	0.6262
Lncident.train^*∗∗*^	**1**	**0.9680**	**0.9749**	**0.9451**	**0.9312**	**0.9289**

Lncident presented the best result on *S. cerevisiae* dataset. ^*∗*^Lncident with model trained on human. ^*∗∗*^The suffix of “train” means the tools with model trained on *C. elegans*.

**Table 4:** Running time on human dataset.

Tools	Running time	
	Stand-alone	Web server^*∗*^
CPC	5 days 4 h 37 min 43 s	About 1 day
CPAT	16 s	
CNCI	37 min 53 s	
PLEK	3 min 04 s	
Lncident	3 min 01 s	About 12 min

Test on Intel® Core™ i7-2600 CPU at 3.40 GHz and 8 GB RAM.

The test dataset contains 4,000 mRNAs and 4,000 lncRNAs. ^*∗*^It is hard to calculate precise running time of testing on web server; thus, we can only obtain a rough approximation. We omitted the result of CPAT web server, because only file size less than 10 MB is allowed, and when we split our file, we found the average error is too serious to accept.
