# Supplementary material for: Lncident: A Tool for Rapid Identification of Long Noncoding RNAs Utilizing Sequence Intrinsic Composition and Open Reading Frame Information
Source: Int J Genomics. 2016 Dec 27;2016:9185496. doi: 10.1155/2016/9185496 (PMC5223071; doi:10.1155/2016/9185496)
Supplement: Supplementary file 1 — This Supplementary Material contains additional information of the original article. In the Supplementary File, a detailed description of datasets and a close comparison of different tools' performances on human/mouse datasets are presented. In addition, the performances of original k-mer scheme (k from 1 to 6) and the performances of different feature group combinations are also included in this material. Finally, we provided top 10 features of Lncident and each feature's importance score, please refer to the Table S2 in Supplementary Material. [file 9185496.f1.docx]

**
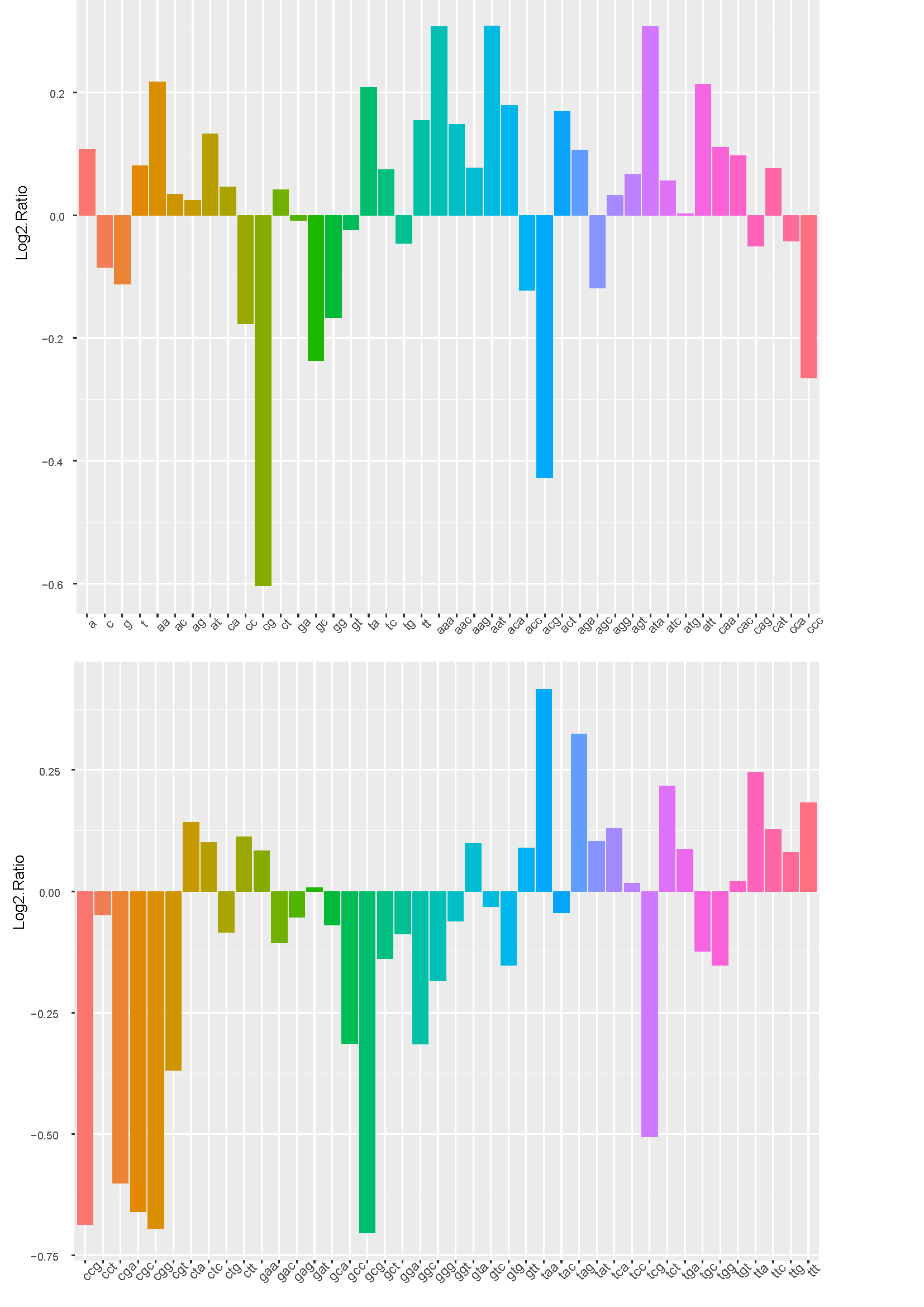
**

**Figure S1. The distribution of the adjoining base(s) on lncRNAs and protein-coding genes** This figure shows the different distribution of adjoining base(s) (*k* = 1, 2, 3) on long non-coding RNAs (lncRNAs) and protein-coding genes. Dataset contains all the lncRNAs and protein-coding genes of the GENCODE database. There are some differences between this figure and Figure 2, which means that the untranslated regions of protein-coding genes indeed have some effects on the performance of classifier. The log_2_ ration in this figure is obtained by calculating log_2_ (adjoining base(s) frequencies of lncRNAs / adjoining base(s) frequencies of protein-coding RNAs).

**
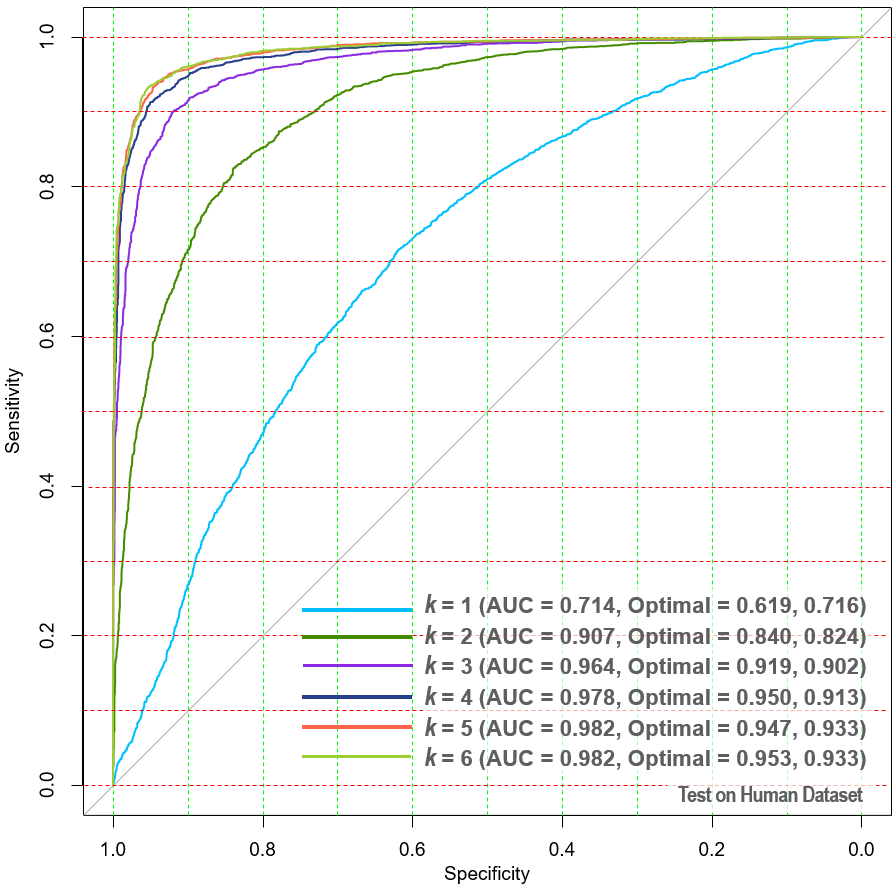
**

**Figure S2. The ROC Curve of k-mer scheme (*k* = 1, 2, 3, 4, 5, 6)** With the increase of *k*, the performance of *k*-mer scheme also gets better. 4,096 values are calculated when *k* = 6, which needs much more time while the result is improved marginally. Thus, we calculate the adjoining base(s) from 1 to 5.


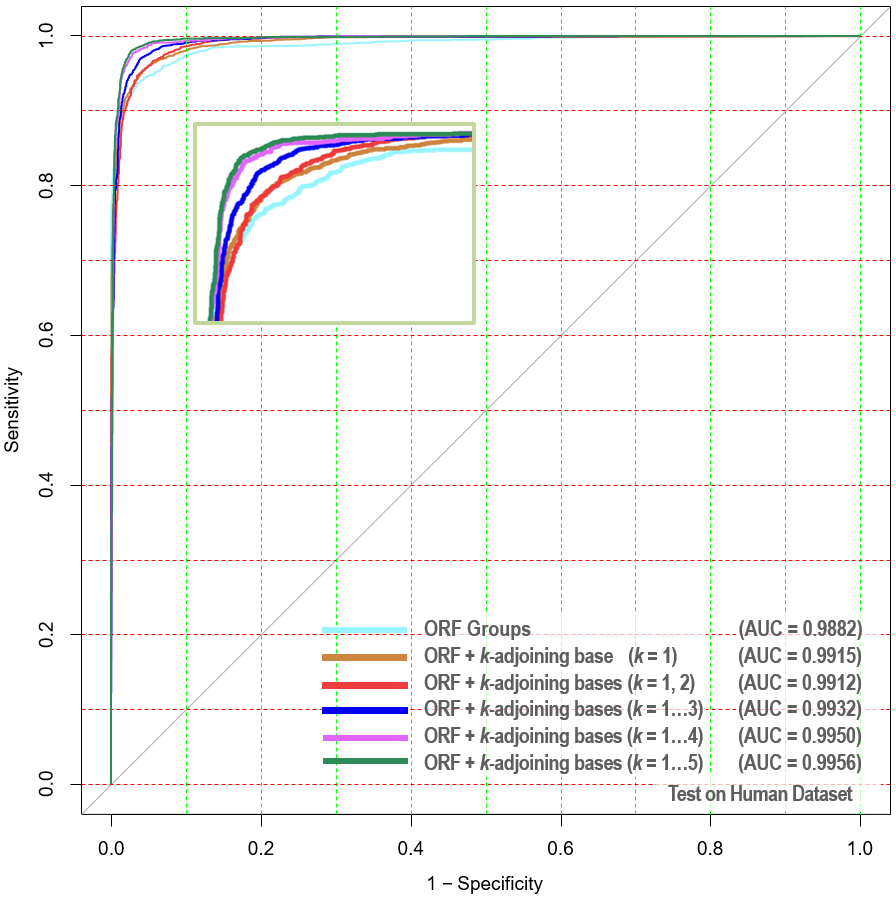


**Figure S3. The ROC Curve of different feature groups combined together** This figure shows the enhancements of new added feature groups. ORF feature group displays a satisfying result.

| **Table S1. Description of datasets** | | |
| --- | --- | --- |
| **Species** | **Database** | **Dataset** |
| **Human 1**  ***(Homo sapiens)*** | GENCODE 24  (GRCh38.p5) | long non-coding RNAs: 28,031  protein-coding transcripts: 28,031 |
| **Human 2**  ***(Homo sapiens)*** | Training set: 8,300 lncRNAs; 20,000 mRNAs  Test set: 4,000 lncRNAs; 4,000 mRNAs | |
| **Mouse**  ***(Mus musculus)*** | GENCODE M9  (GRCm38.p4) | long non-coding RNAs: 13,046  protein-coding transcripts: 13,046 |
| ***Caenorhabditis elegans*** | Ensembl  (WBcel235, Oct 2014) | coding sequences: 24,929 and ncRNAs: 24,929  (lncRNAs: 1,638; small non-coding genes^*^: 23,291) |
| ***Saccharomyces cerevisiae*** | Ensembl  (R64-1-1, Dec 2011) | small non-coding genes genes^*^: 413  coding sequences: 1,500 |
| The dataset of human 1 and mouse were used both as training set of Lncident (10-CV) and test set of all the tools while the dataset of *S. cerevisiae* was only used as test set. For dataset of *C. elegans*, all the sequences were used to evaluate the default model of Lncident, CPC, CPAT, CNCI and PLEK. And 22,929 ncRNAs and 22,929 CDs were selected as training set to re-train CPAT, PLEK and Lncident while the other 2,000 sequences of each class were constructed as test set. All long non-coding RNAs (lncRNAs) or non-coding RNAs (ncRNAs) in the database were included in these four datasets. For dataset of human 2, only one transcript from each gene is collected in training set. In addition, the training and testing datasets have no transcripts from the same genes.  ^*^The ncRNAs of *C. elegans* contains lncRNAs and small non-coding genes. In Ensembl, genes with the following biotypes are classed as small non-coding genes: miRNA, miscRNA, rRNA, scRNA, snlRNA, snoRNA, snRNA, and also the pseudogenic form of these biotypes. | | |

| Table S2. The top 10 features and their importance scores | | | |
| --- | --- | --- | --- |
| Feature | **Importance Score** | **Feature** | **Importance Score** |
| Length of max ORF | 100.00 | CGG | 34.02 |
| Coverage of max ORF | 74.21 | GCGGC | 32.56 |
| CG | 49.72 | CGC | 31.53 |
| CGGCG | 41.22 | CCG | 31.43 |
| GCG | 36.37 | CGGC | 30.48 |
| The scores were calculated based on SVM-RFE algorithm and are normalized according to the highest score, and the most important feature will obtain a score of 100. | | | |

| Table S3. The performances on human species of GENCODE database | | | | |
| --- | --- | --- | --- | --- |
| Tools | **Sensitivity** | **Specificity** | **Accuracy** | **F-measure** |
| CPC | **0.9885** | **0.9985** | **0.9935** | **0.9934** |
| CPAT | 0.9501 | 0.9608 | 0.9555 | 0.9552 |
| CNCI | 0.9734 | 0.9095 | 0.9414 | 0.9433 |
| PLEK | 0.9407 | 0.9448 | 0.9427 | 0.9426 |
| Lncident | 0.9763 | 0.9742 | 0.9752 | 0.9752 |

| Table S4. The performances on mouse species of GENCODE database | | | | |
| --- | --- | --- | --- | --- |
| Tools | **Sensitivity** | **Specificity** | **Accuracy** | **F-measure** |
| CPC | **0.9863** | 0.6861 | 0.8362 | 0.8579 |
| CPAT | 0.9591 | **0.8463** | **0.9027** | **0.9080** |
| CNCI | 0.9633 | 0.8353 | 0.8993 | 0.9055 |
| PLEK | 0.7593 | 0.7709 | 0.7651 | 0.7639 |
| Lncident | 0.9450 | 0.8392 | 0.8921 | 0.8975 |

The results of Table S3, Table S4 are obtained from the average of 10 times predictions. All tools were tested 10 times with the same test sets. All the lncRNAs of human and mouse in GENCODE database are included in the test sets.
